# Psilocybin: the magic medicine for depression?

**DOI:** 10.1192/bjo.2021.456

**Published:** 2021-06-18

**Authors:** Amber Elyse Corrigan, Ella Burchill, Lucy Pelton, Alessia Marrocu, Adele Mazzoleni, Lydia Shackshaft

**Affiliations:** King's College London

## Abstract

**Aims:**

Depression is the single largest contributor to global disability. However, effective treatments are currently lacking, resulting in a significant burden of treatment-resistant depression (TRD). Psilocybin, a serotonergic psychedelic, found as the active compound in 'magic mushrooms', has been proposed as a novel therapeutic avenue for TRD. We aimed to evaluate the future feasibility and implications of psilocybin as a new antidepressant therapy.

**Method:**

We reviewed and critically analysed the available literature on the efficacy and safety of psilocybin as a treatment for depression, and the potential pharmacological and psychological mechanisms of the therapeutic benefit. We discussed the relative contributions to this therapeutic effect of the pharmacological drug treatment, placebo effects, and the context and parameters of the psychotherapeutic experience. We reviewed legal, social, and economic barriers to primary research and clinical implementation.

**Result:**

Psilocybin in combination with psychotherapy has been shown to be safe and effective in TRD. Its mechanism of action in TRD has not been fully elucidated, however reviewing functional neuroimaging studies demonstrated disparate short and long-term modifications of default mode network connectivity, suggested to represent a ‘reset’ mechanism of acute modular disintegration and subsequent reintegration which restores normal function, reviving emotional responsiveness.

Research suggests psychedelic treatment induces lasting personality, belief and attitude changes. The psychedelic drug itself, the context of the psychotherapeutic experience, and the post-drug integration therapy all appear to have a significant role. Preparation prior to treatment, the environment, context and support during the psychedelic experience itself, and the following long-term integration and support process must be considered.

Despite novel findings Psilocybin is a Schedule I drug; this imposes a persisting ethical barrier to clinical use. Prohibition of psilocybin results in high costs of drug supply, and potential for harmful drug-seeking behaviours. Therefore, complex socio-political factors currently limit wider implementation.

**Conclusion:**

Psilocybin in combination with psychotherapy is safe and effective in TRD. The interacting and elusive therapeutic mechanisms have implications for clinical implementation. Preparation prior to treatment, the physical and social environment in which the psychedelic experience takes place, and long-term integration and support are considered to play a significant role. Optimisation of these parameters and cost-benefit analyses are required prior to this being feasible as a widely available therapy. Systemic legislative, political and social change will also be key to enable widespread clinical use. The promise of this therapy on a background of inadequate current antidepressant treatments indicates these must be a priority.

